# Safety profile of enhanced thromboprophylaxis strategies for critically ill COVID-19 patients during the first wave of the pandemic: observational report from 28 European intensive care units

**DOI:** 10.1186/s13054-021-03543-3

**Published:** 2021-04-22

**Authors:** Andrea Lavinio, Ari Ercole, Denise Battaglini, Sandra Magnoni, Rafael Badenes, Fabio Silvio Taccone, Raimund Helbok, William Thomas, Paolo Pelosi, Chiara Robba, collaborators, Nicole Innerhofer, Sara Miori, Alberto Librizzi, Rita Bertuetti, Nicolas Figueiredo Faria, Lorenzo Peluso, Giorgia Montrucchio, Gabriele Sales, Luca Brazzi, Daniela Alampi, Maria Beatrice Manca, Lilia Sepe, Giuseppe Natalini, Antonio Bellino, Maria Grazia Bocci, Chiara Mattana, Francesco Corradi, Francesco Forfori, Francesco Cundari, Emilio Bonvecchio, Zara Busani, Andrea Bianchin, Carla Federico, Anna Santoro, Federico Bilotta, Giorgio Rajani, Berta Moleon Lopez, Raffaele Aspide, Merola Raffaele, Luca Cabrini, Alessandro Motta, Lara Frattini, Alexandre Godon, Pierre Bouzat, Elena Grappa, Alberto Bonvecchio, Nicole Innerhofer, Dietmar Fries, Christian Preuss Hernandez, Claudius Thomé, Sebastian Klein, Michael Joannidis, Paolo Pelosi, Lorenzo Ball, Nicolo’ Patroniti, Iole Brunetti, Matteo Bassetti, Daniele Roberto Giacobbe, Antonio Vena, Alberto Valbusa, Italo Porto, Roberta Della Bona

**Affiliations:** 1 Neurosciences and Trauma Critical Care Unit, Addenbrookes Hospital Cambridge, Cambridge, UK; 2 San Martino Policlinico Hospital, IRCCS for Oncology and Neurosciences, Genoa, Italy; 3 Anestesia e Rianimazione Ospedale Santa Chiara, APSS, Trento, Italy; 4 Department of Anesthesia and Intensive Care, Hospital Clinic Universitari, University of Valencia, INCLIVA Research Health Institute, Valencia, Spain; 5 Department of Intensive Care, Hopital Erasme, Université Libre de Bruxelles, Brussels, Belgium; 6 Department of Neurology, Neurocritical Care Unit, Medical University of Innsbruck, Innsbruck, Austria; 7 Hematology Department, Addenbrookes Hospital, Cambridge, UK; 8 Department of Surgical Sciences and Integrated Diagnostics (DISC), University of Genova, Genoa, Italy; 9 Department of Anaesthesia and Intensive Care, University Hospital Innsbruck, Innsbruck, Austria; 10 Anestesia e Rianimazione, Spedali Civili, Brescia, Italy; 11 Department of Intensive Care Erasme Hospital, Université Libre de Bruxelles, Bruxelles, Belgium; 12 Rianimazione CAR di Città della Salute e della Scienza di Torino, Turin, Italy; 13 Ospedale Sant’andrea Di Roma, Universita Sapienza, Rome, Italy; 14 Department of Anesthesia and Intensive Care, POLIAMBULANZA FOUNDATION, Brescia, Italy; 15 Dipartimento Di Scienze Dell’Emergenza, Anestesiologiche e della Rianimazione Fondazione Policlinico Universitario A. Gemelli IRCCS, Rome, Italy; 16 Department of Anesthesia and Intensive Care, University of Pisa, Pisa, Italy; 17 Anestesia e Rianimazione, Università Degli Studi Di Milano Statale, Milan, Italy; 18 U.O. Anestesia e RianimazioneOspedale S.Valentino Montebelluna, Azienda ULSS 2 Marca Trevigiana, Treviso, Italy; 19 Anestesia e Rianimazione, Città della Salute e della Scienza di Torino, Turin, Italy; 20 Department of Anesthesiology, Critical Care and Pain Medicine, Policlinico Umberto I, “Sapienza” University of Rome, Rome, Italy; 21 Department of Anesthesia and Intensive Care, University of Valencia, Valencia, Spain; 22 IRCCS Istituto Delle Scienze Neurologiche Di Bologna, Anesthesia and Neurointensive Care Unit, Bologna, Italy; 23 Dipartimento Di Scienze Mediche E ChirurgicheAnesthesia and Intensive Care Medicine, Policlinico di Sant’Orsola, Università di Bologna, Bologna, Italy; 24 Presidio Ospedale Di Circolo E Fondazione Macchi, Varese, Italy; 25 Department of Anesthesia and Intensive Care, Grenoble, France; 26 UOS Neuroanestesia, UOC Anestesia e Rianimazione ASST Cremona, Cremona, Italy; 27 Department of General and Surgical Intensive Care Medicine, Medical University Innsbruck, Innsbruck, Austria; 28 Department of Neurosurgery, Medical University Innsbruck, Innsbruck, Austria; 29 Division of Intensive Care and Emergency Medicine, Department of Internal Medicine, Medical University Innsbruck, Anichstraße 35, 6020 Innsbruck, Austria; 30 Department of Health Sciences (DISSAL), University of Genoa, Genoa, Italy; 31 Dipartimento CardioToraco, Vascolare Ospedale Policlinico San Martino IRCCS, Genoa, Italy

**Keywords:** COVID-19, Intensive care medicine, Thrombosis, Heparin, Prophylaxis, Anticoagulation

## Abstract

**Introduction:**

Critical illness from SARS-CoV-2 infection (COVID-19) is associated with a high burden of pulmonary embolism (PE) and thromboembolic events despite standard thromboprophylaxis. Available guidance is discordant, ranging from standard care to the use of therapeutic anticoagulation for enhanced thromboprophylaxis (ET). Local ET protocols have been empirically determined and are generally intermediate between standard prophylaxis and full anticoagulation. Concerns have been raised in regard to the potential risk of haemorrhage associated with therapeutic anticoagulation. This report describes the prevalence and safety of ET strategies in European Intensive Care Unit (ICUs) and their association with outcomes during the first wave of the COVID pandemic, with particular focus on haemorrhagic complications and ICU mortality.

**Methods:**

Retrospective, observational, multi-centre study including adult critically ill COVID-19 patients. Anonymised data included demographics, clinical characteristics, thromboprophylaxis and/or anticoagulation treatment. Critical haemorrhage was defined as intracranial haemorrhage or bleeding requiring red blood cells transfusion. Survival was collected at ICU discharge. A multivariable mixed effects generalised linear model analysis matched for the propensity for receiving ET was constructed for both ICU mortality and critical haemorrhage.

**Results:**

A total of 852 (79% male, age 66 [37–85] years) patients were included from 28 ICUs. Median body mass index and ICU length of stay were 27.7 (25.1–30.7) Kg/m^2^ and 13 (7–22) days, respectively. Thromboembolic events were reported in 146 patients (17.1%), of those 78 (9.2%) were PE. ICU mortality occurred in 335/852 (39.3%) patients. ET was used in 274 (32.1%) patients, and it was independently associated with significant reduction in ICU mortality (log odds = 0.64 [95% CIs 0.18–1.1; *p* = 0.0069]) but not an increased risk of critical haemorrhage (log odds = 0.187 [95%CI − 0.591 to − 0.964; *p* = 0.64]).

**Conclusions:**

In a cohort of critically ill patients with a high prevalence of thromboembolic events, ET was associated with reduced ICU mortality without an increased burden of haemorrhagic complications. This study suggests ET strategies are safe and associated with favourable outcomes. Whilst full anticoagulation has been questioned for prophylaxis in these patients, our results suggest that there may nevertheless be a role for enhanced / intermediate levels of prophylaxis. Clinical trials investigating causal relationship between intermediate thromboprophylaxis and clinical outcomes are urgently needed.

**Supplementary Information:**

The online version contains supplementary material available at 10.1186/s13054-021-03543-3.

## Background

A growing body of observational clinical evidence indicates that coronavirus disease (COVID‐19) is associated with a high incidence of thrombotic complications [1–4]. Despite standard anticoagulant thromboprophylaxis, the burden of thrombotic complications—primarily pulmonary embolism (PE)—remains high in COVID-19 patients, in particular among those requiring intensive care unit (ICU) admission. Recent studies on COVID-19 patients have reported an incidence of thromboembolic events ranging from 27 to 57% [5] despite standard thromboprophylaxis, and a recent review of studies including a total of 1765 hospitalised patients (mixed cohort of patients admitted to the ICU or the ward) reported the occurrence of venous thromboembolism (VTE) in approximately 20% of patients, with cumulative prevalence up to 49% during hospitalisation [6].

Although observational clinical data suggest that the use of either prophylactic to increased doses of low molecular weight heparins (LMWH) in high-risk patients may be associated with better prognosis, the optimal thromboprophylaxis strategy in the critically ill COVID-19 patient population remains uncertain [7]. In the absence of evidence from randomised controlled trials, published guidance based on observational data and expert opinion has been heterogeneous and sometimes contradictory, ranging from standard treatment to a variety of ET protocols with varying levels of anticoagulation from enoxaparin 40 mg BD to full therapeutic anticoagulation with unfractionated heparin [8–11]. Recently, three randomised clinical trials aimed to test the effects of full doses of anticoagulants in COVID-19 patients have paused enrolment for futility, questioning the benefit of giving full dose anticoagulants routinely in critically ill COVID-19 patients and raising concerns regarding the safety of widespread ET protocols [12].

The purpose of this study was therefore to describe the prevalence of ET strategies in European Intensive Care Unit (ICUs) and to assess their association with ICU mortality and safety in a large cohort of critically ill COVID-19 patients admitted to European ICUs during the first wave of the pandemic.

## Methods

### Study population and data collection

This is an observational, retrospective multi-centre study, including 28 European ICUs. The study was conducted according to the Strengthening the Reporting Observational Studies in Epidemiology (STROBE) Statement guidelines (Supplementary material Section S1). Each participating centre obtained the approval for data collection from its local Ethical Committee. The need for written informed consent was waived for retrospectively collected data. In each centre, consecutive adult patients (age ≥ 18) with a confirmed COVID-19 diagnosis requiring ICU admission in the period between 26 February and 30 May 2020 were included. Participating units were provided with a study protocol and case report form (CRF). A detailed list of all variables collected in the CRF is provided in supplementary material (section S6). For each centre a trained physician collected, curated and submitted anonymised data for analysis to the coordinating centre (Cambridge, UK). Analysis was performed by AE, CR and AL. Data included patient demographics [age; gender; weight; body mass index, BMI], past medical history [hypertension, diabetes, renal failure, cardiac failure, renal failure], date of ICU admission and discharge, ICU mortality, mode of death [respiratory failure, multiorgan failure, major haemorrhage, cardiocirculatory collapse], renal failure [acute kidney injury (AKI), need for renal replacement therapy (RRT)], thromboembolic events [i.e. pulmonary embolism (PE), deep venous thrombosis (DVT), arterial embolism, line clotting] and haemorrhagic events [critical (i.e. intracranial bleed or requiring transfusion), non-critical (i.e. others)]. Data regarding any antiplatelet therapy, thromboprophylaxis and therapeutic anticoagulation at ICU admission were also obtained. Participating centres were requested to indicate whether patients were treated with standard prophylaxis (group *‘standard prophylaxis’*) or COVID-19 specific ‘enhanced thromboprophylaxis’ according to local protocols (group *‘ET’*) and which molecule was used (presented as two subgroups *‘ET enoxaparin’* and *‘ET other’*). Authors were asked to describe local protocols, including whether anti-Xa monitoring was routinely used for titration. Patients already on therapeutic anticoagulation at the time of ICU admission for established or suspected thromboembolic events were included in the group *‘therapeutic anticoagulation for indications other than prophylaxis’*.

### Statistical analysis

**Fig. 1:**
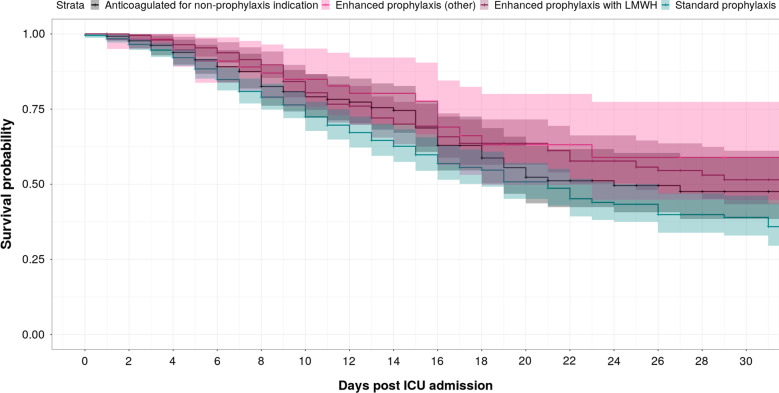
Kaplan–Meier plot stratified by anticoagulation status: Standard prophylaxis, enhanced prophylaxis with LMWH (light purple), enhanced prophylaxis with other agent (darker purple), anticoagulated for non-prophylaxis indication. In this unmatched analysis, a trend towards improved survival with enhanced prophylaxis (purple curves) is apparent

Statistical analysis was performed in R 3.6.3 [13]. The data collection and curation process are described in the DAQCORD statement given in supplementary materials S2 [14]. Kaplan–Meier ICU survival curve plot for the various anticoagulation groups was performed (Fig. 1). Modelling was undertaken using the lme4 v1.1-25 [15], MICE v3.11.0 [16] and MatchIt v4.0.0 [17] packages. Statistical significance was taken as *p* < 0.05, and corrections for multiple comparisons were not made. Multiple imputations using a predictive mean matching method on all variables were used to generate 50 complete datasets. Outcomes were included in the imputation but subjects with missing outcomes excluded from the final matching for statistical efficiency. For each imputed dataset, propensity scores for receiving ET for thromboprophylaxis were estimated using a mixed effects model including age, BMI, medical history (presence/absence of hypertension, diabetes or renal disease), D-dimers on admission, C-reactive protein, fibrinogen, platelet count, white blood cell count, intubation status and antiplatelet agent use, as fixed effects and site code as a random effect. Propensity matched datasets were then constructed using a nearest neighbour approach before fitting additional mixed effects models for ICU survival and for, with the same covariates and random effects on each of these matched datasets. The final results were then pooled.

## Results

### Study population

Data completeness was good and is summarised in supplementary materials (Additional file 1: section S3). Anonymised data for 852 patients were provided by 28 collaborating European sites. Patient characteristics are summarised in Table 1. Six hundred and seventy-seven (79.5%) were male, and median age was 66 [37–85] years. Hypertension and obesity were the most common comorbidities, occurring in the 52.3% and 27.6% of patients, respectively. Median body mass index (BMI) was 27.7 [25.1–30.7] Kg/m^2^.

**Table 1:** Demographics, blood tests results at intensive care unit (ICU) admission and during ICU stay, outcomes and complications of the overall population and according to different subgroups

	Overall (*N* = 852)	Enhanced thromboprophylaxis [enoxaparin] (*N* = 236, 27.7%)	Enhanced thromboprophylaxis [other] (*N* = 38, 4.5%)	Standard prophylaxis (*N* = 435, 51%)	Anticoagulation for indication other than prophylaxis [i.e. PE / DVT on admission] (*N* = 143, 16.8%)
Sex, male, n (%)	677 (79.5)	187 (79.2)	31 (81.6)	340 (78.2)	119 (83.2)
Age, years	66 (37–85)	66 (37–85)	65.5 (37–86)	66 (16–87)	67 (27–85)
*Comorbidities, n (%)*					
Hypertension	446 (52.3)	127 (53.8)	16 (42.1)	217 (49.9)	57 (39.9)
Diabetes mellitus	143 (16.8)	37 (15.7)	1 (2.6)	79 (18.2)	26 (18.2)
Renal disease	44 (5.2)	14 (5.9)	0 (0.0)	29 (6.7)	1 (0.7)
Cardiac dysfunction	86 (10.1)	26 (11)	1 (2.6)	41 (9.4)	18 (12.6)
Liver disease	16 (1.9)	2 (0.8)	2 (5.2)	10 (2.3)	2 (1.4)
Obesity (BMI > 30 kg/m^2^)	235 (27.6)	72 (30.5)	10 (26.3)	111 (25.5)	42 (29.4)
*Bloods at ICU admission, median (IQR)*					
WBC, cells × 10^9^/L	9.0 (1–89)	10.6 (1–46)	9.4 (3–18)	9.0 (2–89)	9.1 (3–45)
D-dimer, ng/mL	1340 (150–136,076)	1610 (93–105,990)	2291 (180–76,400)	1207 (150–129,064)	1484 (85–136,076)
Platelets, cells^3^/µL	223 (31–734)	218 (255–654)	219 (133–517)	218 (200–734)	234 (70–814)
Fibrinogen, mg/dL	637 (77–1323)	613 (100–1276)	649 (163–999)	635 (40–1196)	626.5 (77–1323)
C-reactive protein, mg/L	102.3 (1–559)	146 (0–559)	162 (3–387)	136.5 (0–393)	138.5 (1–255)
Troponin-I, ng/mL	0.02 (0–6)	0.02 (0–2)	0.03 (0–0)	0.02 (0–21)	0.02 (0–3)
Creatinine, mg/dL	0.93 (0–7)	0.82 (0–7)	1 (1–3)	0.95 (0–7)	0.9 (0–8)
PaO_2_, mmHg	80 (25–440)	76.5 (27–316)	67.5 (34–189)	85 (25–440)	83 (39–489)
*Bloods during ICU stay, median (IQR)*					
WBC, cells × 10^9^/L					
Lowest	6.1 (0–44)	6.0 (1–24)	6.3 (2–18)	6.1 (1–31)	5.9 (1–44)
Highest	17.5 (2–132)	17.8 (2–132)	15.3 (2–31)	16.8 (3–68)	19.6 (5–80)
D-dimer, ng/mL					
Highest	4395 (176–222,032)	4706.5 (635–222,032)	6320 (703–798,94)	3637.5 (201–57,588)	5273.5 (201–57,588)
Platelets, cells^3^/µL					
Lowest	169 (110–510)	174 (30–315)	189 (91–476)	173 (18–510)	146 (11–476)
Highest	380 (700–981)	397 (175–981)	417 (154–645)	379 (118–953)	366 (70–645)
Troponin-I, ng/mL					
Highest	0.04 (0–10)	0.03 (0–2)	0.03 (0–0)	0.03 (0–10)	0.05 (0–5)
Creatinine, mg/dL					
Highest	1.4 (0.4–14)	1.3 (0–12)	1.2 (1–6)	1.4 (0–9)	1.6 (1–14)
PaO_2_, mmHg					
Lowest	61 (26–150)	61 (30–107)	60.9 (34–88)	61 (26–150)	62 (33–130)
Thromboembolic complications* n (%)					
Arterial embolism	8 (0.9)	6 (2.5)	0 (0.0)	2 (0.5)	0 (0)
DVT	28 (3.2)	11 (4.6)	0 (0.0)	17 (3.9)	0 (0)
Line clotted	21 (2.4)	5 (2.1)	1 (2.6)	15 (3.4)	0 (0)
Pulmonary embolism	57 (6.6)	21 (8.8)	4 (10.5)	32 (7.4)	0 (0)
No/NA	738 (86.6)	193 (82.0)	33 (86.9)	369 (84.8)	0 (0)
*Haemorrhagic complications n (%)*					
Critical haemorrhage	47 (5.5)	12 (5.0)	0 (0.0)	27 (6.2)	8 (5.6)
Non-critical haemorrhage	58 (6.8)	16 (6.9)	2 (5.3)	28 (6.4)	12 (8.4)
No/NA	747 (87.7)	208 (88.1)	36 (94.7)	380 (87.4)	123 (86.0)

IQR, interquartile range; BMI, body mass index; WBC, white blood cells; PaO_2_,partial pressure of oxygen; AKI, acute kidney injury; RRT, renal replacement therapy; DVT, deep venous thrombosis; PE, pulmonary embolism; NA, not available. * after ICU admission and initiation of anticoagulant regimen

Median distribution of BMI in the enhanced prophylaxis group did not differ from those in the rest of the patients (*p* = 1) (Additional file 1: section S4, Figure S2). Distribution of ICU admission fibrinogen, platelet count, prothrombin time, D-dimer C-reactive protein and white blood cell count in the enhanced prophylaxis group compared to the rest of the patients showed no difference in median values (Wilcoxon rank sum all *p* = 1) (Additional file 1: section S4, Figure S3,4). Details on ICU complications, bloods and therapy according to the different groups are presented in Table 1.

Median length of ICU stay was 13 [7–22] days. ICU survival status was available for 816 patients: 337 of those died (41.3%). In non-survivors, the reported mode of death was multiorgan failure 172 (51%), cardiocirculatory collapse 87 (25.8%), respiratory failure 73 (21.7%) and massive haemorrhage in 3 (0.9%). Of the three deceased patients for whom the reported mode of death was massive haemorrhage, one patient was being treated with ET and 2 patients were on standard thromboprophylaxis regimens.

### Thromboembolic events and anticoagulation regimens

A total of 274 (32.2%) patients received *enhanced thromboprophylaxis (ET)* according to local protocols. The majority 236 (27.7%) received ET with enoxaparin (group *‘ET enoxaparin’*) at doses reported ranging from 100 to 200 IU/Kg/day in two divided doses (i.e. equivalent to approximately 40–80 mg twice daily for an 70–90 kg adult), with correction for renal failure and bleeding abnormalities during the course of ICU stay according to local practice.

Only one centre (Bruxelles) reported using anti-Xa activity with a target of (0.3–0.5) systematically for dose titration. Thirty-eight (4.5%) patients (group ‘*ET other*’) received ET with UFH (38) titrated to heparin ratio of 1.5–2.5, or fondaparinux (one patient).

Four hundred and thirty-five (51.1%) received thromboprophylaxis as per standard protocols (group *‘standard prophylaxis’*). These include 19 (2.2%) patients with contraindications to anticoagulation at the time of ICU admission who received no heparin. Indications for anticoagulation for indication other than prophylaxis (143 cases, 16.8%) were arterial embolism (1 case, 0.7%), deep venous thrombosis (3 cases, 2.1%), line clotted (7 cases, 4.9%) and pulmonary embolism (21 cases, 14.7%).

Thromboembolic events after ICU admission were reported in 114 patients (13.3%), including 57 (6.6%) cases of pulmonary embolism. A crude comparison between ET and standard prophylaxis (after excluding for patients with indications for therapeutic anticoagulation other than prophylaxis at time of ICU admission) does not reveal a statistical difference in reported thromboembolic events (*p* = 0.4).

### Predictors of outcome and critical haemorrhage

Figure 1 shows a Kaplan–Meier ICU survival curve plot for the various anticoagulation groups; a trend towards improved survival with enhanced prophylaxis is apparent. The results of the propensity score analysis for ICU mortality are summarised in Table 2. A control match was found for each of the patients treated with ET. The use of ET was independently associated with significant reduction in ICU mortality (log odds = 0.64 [95% CIs 0.18–1.1; *p* = 0.0069]) but not an increased risk of critical haemorrhage (log odds = 0.187 [95%CI − 0.591 to − 0.964; *p* = 0.64]). Older age and high BMI were found to be associated with a higher log odds of ICU mortality (log odds = −12.1 [95% CI − 15.6 to − 8.69; *p* < 0.0001] and −1.34 [95% CI − 2.46 to − 0.211; *p* = 0.02], respectively). Increased ICU admission platelet count was associated with increased log odds of ICU mortality [log odds = 61.5 [95% CI 27.7–95.4; *p* = 0.0004]) as was the use of mechanical ventilation [log odds = 2.28 [95% CI 1.1–3.5; *p* = 0.00026]. There were no other statistically significant predictors of ICU mortality in the multivariate model.

**Table 2:** Mixed effects, generalised linear model for ICU survival matched for propensity for use of ‘enhanced’ prophylaxis. Effect sizes are unscaled log odds (positive indicates survival benefit)

Term	Effect size (log odds)	*p*-value	95% CI	
(Intercept)	0.38	0.37	− 0.452	1.21
Use of ‘enhanced’ (therapeutic) prophylaxis	0.64	0.0069	0.176	1.1
Age (years)	− 12.1	< 0.0001	− 15.6	− 8.69
BMI	− 1.34	0.02	− 2.46	− 0.211
History of hypertension	− 0.0204	0.94	− 0.53	0.489
History of diabetes	− 0.07	0.83	− 0.70	0.563
History of renal disease	− 0.951	0.1	− 2.1	0.198
Intubated	2.28	0.00026	1.1	3.5
D-dimer at ICU_admission	− 3390	0.38	− 11,000	4180
P/F ratio at ICU admission	18.4	0.13	− 5.24	42
CRP at ICU admission	− 19.8	0.26	− 54.3	14.7
Fibrinogen_at ICU_admission	− 5.14	0.9	− 89.8	79.5
Platelet count at_ICU_admission	61.5	0.0004	27.7	95.4
WBC at ICU admission	− 1.73	0.052	− 3.46	0.0119
Antiplatelet agent use	0.44	0.2	− 0.238	1.12

BMI, body mass index; ICU, intensive care unit; P/F partial pressure of oxygen/inspired fraction of oxygen; CRP, C-reactive protein; WBC, white blood cells

Table 3 shows the results of the propensity matched analysis for critical haemorrhage. There were no statistically significant predictors of critical haemorrhage in our dataset. Most importantly, ET was not significantly associated with an increased risk of critical haemorrhage (log odds = 0.187 [95%CI − 0.591 to − 0.964; *p* = 0.64]).

**Table 3:** Mixed effects, generalised linear model for occurrence of ‘critical haemorrhage’ (intracranial haemorrhage or haemorrhage requiring transfusion matched for propensity for use of ‘enhanced’ prophylaxis

	Effect size (log odds)	*p* value	95% CI	
(Intercept)	− 3.31	< 0.0001	− 4.41	− 2.21
Use of ‘enhanced’ (therapeutic) prophylaxis	0.187	0.64	− 0.591	0.964
Age (years)	− 3.57	0.1	− 7.85	0.713
BMI	− 0.83	0.41	− 2.79	1.13
History of hypertension	− 0.0719	0.86	− 0.879	0.736
History of diabetes	− 0.341	0.55	− 1.47	0.792
History of renal disease	− 0.374	0.73	− 2.52	1.77
Intubated	− 0.757	0.38	− 2.44	0.931
D-dimer at ICU_admission	− 7960	0.33	− 24,000	8070
P/F ratio at ICU admission	8.38	0.62	− 24.6	41.3
CRP at ICU admission	27.7	0.32	− 27	82.4
Fibrinogen at ICU_admission	− 89.9	0.14	− 210	30.2
Platelet count at_ICU_admission	2.71	0.91	− 44.6	50
WBC at ICU admission	2.11	0.039	0.11	4.12
Antiplatelet agent use	0.666	0.2	− 0.358	1.69

Effect sizes are unscaled log odds (positive indicates associate with increased risk of critical haemorrhage)

BMI, body mass index; ICU, intensive care unit; P/F partial pressure of oxygen/inspired fraction of oxygen; CRP, C-reactive protein; WBC, white blood cells

A sensitivity analysis was performed, repeating both propensity models but excluding all patients who received full anticoagulation for non-prophylaxis indications as these might ‘enrich’ the standard group with patients with higher risk of mortality due to significant thromboembolic disease, biasing the results against standard treatment. However, the results were qualitatively the same and revealed identical statistically significant associations (see Additional file 1: section S5) suggesting that our analysis is robust.

## Discussion

We report on the wide adoption of empirically *‘enhanced'* thromboprophylaxis strategies for critically ill COVID-19 patients during the first wave of the pandemic. These enhanced strategies varied among European centres. The most common strategy consisted in increasing LMWH prophylaxis to an intermediate range between standard prophylaxis and full therapeutic anticoagulation. A minority of centres opted for full therapeutic anticoagulation with unfractionated heparin.

The main finding of this study is that the introduction of ‘enhanced thromboprophylaxis’ strategies was not associated with an increased incidence of haemorrhagic events and it was associated with increased ICU survival in propensity matched analysis.

These findings are of particular relevance in view of the recent suspension on the grounds of futility for three clinical trials investigating the effects of full doses of anticoagulants in critically ill COVID-19 patients [12].

The association of intermediate ‘enhanced thromboprophylaxis’ strategies with the improved survival in the absence of increased haemorrhagic complications suggests that standard approaches may safely be augmented. Whilst caution needs to be employed given the non-random allocation and variances in practice and we do not claim statistical significance, the survival curve of Fig. 1 is consistent with the improved survival with ET found in the propensity matched model.

Dysregulated coagulation, systemic prothrombotic state and local micro-thrombosis associated with acute endothelial inflammation, hypoxia, apoptosis and platelet activation are the main pathophysiological mechanisms underlying COVID-19-related coagulopathy [1–4].

There is fairly convincing evidence that in situ pulmonary artery microthrombi may partly represent the endpoint of pulmonary inflammation [6, 19].

Based on such aetiological considerations, it would seem reasonable to assume that therapeutic interventions should primarily target the early stages of the process (i.e. inflammation modulation and inhibition of platelet activation) rather than the coagulation cascade (thus discounting the potential benefits of heparin-based treatments) [20]. Moreover, if microvascular coagulation occurs as a manifestation of end-stage lung inflammation, alveolar damage and hypoxia (i.e. pulmonary thrombosis seen as a tombstone rather than a risk factor for cardiocirculatory collapse, respiratory failure and fatal outcome), then anticoagulation or thrombolysis would incur the risk of precipitating pulmonary haemorrhage without proving any benefit [20].

Whilst it is not disputed that immunomodulation and inhibition of platelet activation are certainly key targets for the care of critically ill COVID-19 patients [21] (dexamethasone is the only drug clearly proven to reduce mortality at the time of writing [20]), our results support the use of ET strategies. Although the mechanisms of heparin resistance in critically ill COVID-19 patients remain to be fully elucidated, the phenomenon has been clearly described and could be at least partially attributed to high factor VIII and fibrinogen and low antithrombin levels typically seen in these patients [23]. Heparin resistance with unfractionated heparin or sub-optimal anti-Xa peak with low molecular weight heparin was confirmed to be a common occurrence. It was furthermore confirmed that in vitro spiking of COVID-19 samples from patients in intensive care unit with low molecular weight heparin failed to recover the anti-Xa level as would have been predicted [24]. In conjunction with the evidence of high rate of thromboembolic events despite standard thromboprophylaxis, the evidence of heparin resistance supports the implementation of increased prophylactic dosing in critically ill COVID-19 patients [21].

Furthermore, preliminary studies reported a significant reduction in thromboembolic events for critically ill COVID-19 patients treated with empirical ET strategies when compared to standard prophylaxis (*N* = 26, 56% vs 100%, *p* = 0.03) [22]. These findings replicate earlier experience in patients developing ARDS secondary to influenza A [H1N1], where empirical ‘therapeutic’ heparin prophylaxis was associated with a 33-fold reduction in thromboembolic events, crucially in the absence of increased haemorrhagic complications [23].

Massive pulmonary embolism may be a potentially reversible cause of death and therefore a potential therapeutic target in critically ill COVID-19 patients. A case series of post-mortem autopsies found that venous thromboembolism was present in 7 of 12 (58%) patients with COVID-19. The study concluded that pulmonary embolism had been the direct cause of death in a third of cases [15]. This is consistent with our findings of a high prevalence of pulmonary embolism and sudden cardiocirculatory collapse and respiratory failure as the most prevalent modes of death. Whether this process can be prevented or reversed remains to be proven, but in a series of three patients with severe COVID-19 respiratory failure who were treated with tissue plasminogen activator a temporally related improvement in respiratory status was reported in all cases (with one of them being a durable response) suggesting a potential reversibility of the process [24].

### Risk of haemorrhage

A French single centre study on 92 critically ill COVID-19 patients reported a 40% prevalence of thromboembolic events (TE) and a 21% rate of ‘significant’ thromboembolic events, with most of such events occurring in patients being treated with full dose anticoagulation. The authors concluded: *“as half of these patients were treated with full-dose pre-emptive anticoagulation without a confirmed TE, we must be cautious about our thromboprophylaxis strategy with daily reassessment of its indication”* [25]. Whilst we echo the call for caution, the findings of our study seem to indicate that the use of ET is *not* associated with an increased chance of death or critical haemorrhagic events.

### Practical considerations

Given the high burden of thromboembolic complications associated with standard prophylaxis and the absence of major haemorrhage related mortality, the implementation of ‘enhanced’ thromboprophylaxis strategies seems justified. Whilst the ideal dosing and stratification remains to be determined by randomised clinical trials, the implementation of twice daily standard LMWH prophylaxis appears to be reasonable and has the advantage of limiting staff exposure when compared to continuous UFH infusion and aPTT monitoring. In view of the prevalence of renal impairment in this patient population, careful dose adjusting and anti-Xa and aPTT-ratio monitoring is strongly recommended. Given the high prevalence of thromboembolic events even in the absence of risk factors [26] and in consideration of limited validation for risk stratification tools, the authors support a standard ‘universal’ approach to ‘enhanced thromboprophylaxis’ for critically ill COVID-19 patients.

### Limitations of the study

Our study also has several important limitations. Firstly, being a retrospective observational dataset, no definite conclusions can be taken in regard to what the ideal thromboprophylaxis strategy for critically ill COVID-19 patients should be as it is impossible to be sure that the propensity score captures the true decision making in instituting ET in an observational dataset. Secondly, as this is an observational and not interventional study, each centre relied on its own screening methods for the detection of thromboembolic complications, without a systematic screening of patients for haemorrhagic and thrombotic events. Also, we limited our observations and anticoagulant therapy at admission and in the early phases of ICU admission, thus reducing the potential effect of long-term anticoagulant strategies. Moreover, the multicentric nature of the study could potentially increase variance and data integration difficulties among different centres, which is simply not possible to correct by means of post hoc analysis. Whilst clearly not as robust in demonstrating causality as a well conducted randomised controlled trial, our propensity score method attempts to exploit ‘natural’ variations in practice within and between sites to remove bias from the ET cohort.

## Conclusions

Enhanced thromboprophylaxis strategies have been widely and empirically implemented across European ICUs during the first wave of the pandemic. Thromboembolic events remain highly prevalent. Death associated with massive haemorrhage is extremely rare, and it does not appear to be associated with ‘enhanced thromboprophylaxis’ strategies, which in this series consisted in increasing low molecular weight heparin within an intermediate range between standard prophylactic and full therapeutic dose. Within the limitations of its methodology, this study supports the continued use of enhanced intermediate levels of thromboprophylaxis for critically ill COVID-19 patients. Further well designed randomised controlled trials are urgently needed to explore the causal relationship between the dose of anticoagulation received and patients’ outcome in critically ill COVID-19 patients.

## Supplementary Information

**Additional file 1:** Supplementary statistical analysis and results.

## Data Availability

The data are fully available, please contact the corresponding author.

## Abbreviations

ET: Enhanced thromboprophylaxis
ICU: Intensive care unit
COVID‐19: Coronavirus disease
PE: Pulmonary embolism
VTE: Venous thromboembolism
LMWH: Low molecular weight heparin
aPTT: Activated partial thromboplastin time
DVT: Deep venous thrombosis
BMI: Body mass index
ICU: Intensive care unit
P/F: Partial pressure of oxygen/inspired fraction of oxygen
CRP: C-reactive protein
WBC: White blood cells
