# Supplementary material for: Safety profile of enhanced thromboprophylaxis strategies for critically ill COVID-19 patients during the first wave of the pandemic: observational report from 28 European intensive care units
Source: Crit Care. 2021 Apr 22;25:155. doi: 10.1186/s13054-021-03543-3 (PMC8061459; doi:10.1186/s13054-021-03543-3)
Supplement: Supplementary file 1 — Additional file 1: Supplementary statistical analysis and results. [file 13054_2021_3543_MOESM1_ESM.pdf]

# SUPPLEMENTARY MATERIAL

## Safety profile of enhanced thromboprophylaxis strategies for critically ill COVID-19 patients during the first wave of the pandemic: observational report from 28 European intensive care units.

Andrea Lavinio[1], Ari Ercole[1], Denise Battaglini[2], Sandra Magnoni[3], Rafael Badenes[4], Fabio Silvio Taccone[5], Raimund Helbolk[6], William Thomas[7], Paolo Pelosi [2,8], Chiara Robba[2,8] and collaborators*

[1]Neurocritical Care Unit, Addenbrookes Hospital Cambridge, UK
[2]Policlinico San Martino, IRCCS for Oncology and Neuroscience, Genova, Italy
[3] Anestesia e Rianimazione Ospedale Santa Chiara APSS – Trento, Italy
[4] Department of Anesthesia and Intensive Care,University of Valencia, Spain
[5]Department of Intensive Care, Hopital Erasme, Université Libre de Bruxelles, Brussels, Belgium
[6]Department of Neurology, Neurocritical Care Unit, Medical University of Innsbruck, Innsbruck, Austria
[7]Hematology Department, Addenbrookes Hospital, Cambridge
[8]Dipartimento di Scienze Chirurgiche e Integrate, University of Genova, Italy

Sections:

S1 STROBE statement
S2 Data quality: DAQCORD analysis
S3 Data quality: missingness analysis
S4 Distributions for enhanced prophylaxis group
S5 Propensity score sensitivity analysis
S6 Data dictionary / Full list of variables collected

## STROBE Statement—checklist of items that should be included in reports of observational studies

| | Item No | Recommendation | Page No |
|---|---|---|---|
| **Title and abstract** | 1 | (*a*) Indicate the study's design with a commonly used term in the title or the abstract | 1 |
| | | (*b*) Provide in the abstract an informative and balanced summary of what was done and what was found | 8,9 |
| **Introduction** | | | |
| Background/rationale | 2 | Explain the scientific background and rationale for the investigation being reported | 10 |
| Objectives | 3 | State specific objectives, including any prespecified hypotheses | 11 |
| **Methods** | | | |
| Study design | 4 | Present key elements of study design early in the paper | 12 |
| Setting | 5 | Describe the setting, locations, and relevant dates, including periods of recruitment, exposure, follow-up, and data collection | 12 |
| Participants | 6 | (*a*) *Cohort study*—Give the eligibility criteria, and the sources and methods of selection of participants. Describe methods of follow-up<br>*Case-control study*—Give the eligibility criteria, and the sources and methods of case ascertainment and control selection. Give the rationale for the choice of cases and controls<br>*Cross-sectional study*—Give the eligibility criteria, and the sources and methods of selection of participants | 12 |
| | | (*b*) *Cohort study*—For matched studies, give matching criteria and number of exposed and unexposed<br>*Case-control study*—For matched studies, give matching criteria and the number of controls per case | |
| Variables | 7 | Clearly define all outcomes, exposures, predictors, potential confounders, and effect modifiers. Give diagnostic criteria, if applicable | 12-13 |
| Data sources/ measurement | 8* | For each variable of interest, give sources of data and details of methods of assessment (measurement). Describe comparability of assessment methods if there is more than one group | *12* |
| Bias | 9 | Describe any efforts to address potential sources of bias | 13 |
| Study size | 10 | Explain how the study size was arrived at | 12 |
| Quantitative variables | 11 | Explain how quantitative variables were handled in the analyses. If applicable, describe which groupings were chosen and why | 13 |
| Statistical methods | 12 | (*a*) Describe all statistical methods, including those used to control for confounding | 13 |
| | | (*b*) Describe any methods used to examine subgroups and interactions | 13 |
| | | (*c*) Explain how missing data were addressed | 13 |
| | | (*d*) *Cohort study*—If applicable, explain how loss to follow-up was addressed<br>*Case-control study*—If applicable, explain how matching of cases and controls was addressed<br>*Cross-sectional study*—If applicable, describe analytical methods taking account of sampling strategy | 13 |
| | | (*e*) Describe any sensitivity analyses | |

Continued on next page

| | | | |
|---|---|---|---|
| **Results** | | | |
| Participants | 13* | (a) Report numbers of individuals at each stage of study—eg numbers potentially eligible, examined for eligibility, confirmed eligible, included in the study, completing follow-up, and analysed | 14 |
| | | (b) Give reasons for non-participation at each stage | |
| | | (c) Consider use of a flow diagram | |
| Descriptive data | 14* | (a) Give characteristics of study participants (eg demographic, clinical, social) and information on exposures and potential confounders | 14 |
| | | (b) Indicate number of participants with missing data for each variable of interest | 14,15 |
| | | (c) *Cohort study*—Summarise follow-up time (eg, average and total amount) | 14,15 |
| Outcome data | 15* | *Cohort study*—Report numbers of outcome events or summary measures over time | 14,15 |
| | | *Case-control study*—Report numbers in each exposure category, or summary measures of exposure | 14,15 |
| | | *Cross-sectional study*—Report numbers of outcome events or summary measures | 14,15 |
| Main results | 16 | (*a*) Give unadjusted estimates and, if applicable, confounder-adjusted estimates and their precision (eg, 95% confidence interval). Make clear which confounders were adjusted for and why they were included | 14,15 |
| | | (*b*) Report category boundaries when continuous variables were categorized | 14,15 |
| | | (*c*) If relevant, consider translating estimates of relative risk into absolute risk for a meaningful time period | 14,15 |
| Other analyses | 17 | Report other analyses done—eg analyses of subgroups and interactions, and sensitivity analyses | 14,15 |
| **Discussion** | | | |
| Key results | 18 | Summarise key results with reference to study objectives | 16 |
| Limitations | 19 | Discuss limitations of the study, taking into account sources of potential bias or imprecision. Discuss both direction and magnitude of any potential bias | 19-20 |
| Interpretation | 20 | Give a cautious overall interpretation of results considering objectives, limitations, multiplicity of analyses, results from similar studies, and other relevant evidence | 16-17 |
| Generalisability | 21 | Discuss the generalisability (external validity) of the study results | 16-17 |
| **Other information** | | | |
| Funding | 22 | Give the source of funding and the role of the funders for the present study and, if applicable, for the original study on which the present article is based | 20 |

*Give information separately for cases and controls in case-control studies and, if applicable, for exposed and unexposed groups in cohort and cross-sectional studies.

## S2. Data quality: DAQCORD statement

The data design, collection and curation is described in the DAQCORD analysis below.

| Indicator | |
|---|---|
| 1. The case report form (CRF) has been designed by a team with a range of expertise. | The data collection was designed by the senior authors who are qualified intensive care medical practitioners with substantial experience in the treatment of COVID-19 patients. |
| 2. There is a robust process for choosing and designing the data set to be collected that involves appropriate stakeholders, including a data curation team with appropriate skill mix. | As per point 1, design involved informal consensus amongst the senior authors |
| 3. The data ontology is consistent with published standards (common data elements) to the greatest extent possible. | No ontology needed in this case as this was a snapshot observational study of clinical, demographic and laboratory parameters |
| 4. Data types are specified for each variable. | This was not enforceable up front due to time constraints preventing the setup of a formal eCRF. However, data types were enforced at data curation. |
| 5. Variables are named and encoded in a way that is easy to understand. | Variable names were all descriptive. |
| 6. Relational databases have been appropriately normalised: steps have been taken to eliminate redundant data and remove potentially inconsistent or overly complex data dependencies. | Not used in this case |
| 7. Each individual has a unique identifier. | Anonymous identifiers were used. |
| 8. There is no duplication in the data set: data have not been entered twice for the same participant. | This is possible but very unlikely as the data was gathered by clinicians on case review. |
| 9. Data that are mandatory for the study are enforced by rules at data entry, and user reasons for overriding the error checks (queries) are documented in the database. | This was not enforceable up front due to time constraints preventing the setup of a formal eCRF. However, data types were enforced at data curation. |
| 10. Missingness is defined and is distinguished from “not available”, “not applicable”, “not collected” or “unknown”. For optional data, “not entered” is differentiated from “not clinically available” depending on research context. | These logical categories were equivalent for the variables in this study as all data should have been part of routine care: Thus, if it was missing it should be at random although conceivably might be a soft indicator of site performance. |
| 11. Range and logic checks are in place for CRF response fields that require free entry of numeric values. Permissible values and units of measurement are specified at data entry. | This was not enforceable up front due to time constraints preventing the setup of a formal eCRF. Range and sense checking were undertaken at data curation before analysis. |
| 12. Free text avoided unless clear scientific justification and (e.g. qualitative) analysis plan specified and feasible. | This was not enforceable up front due to time constraints preventing the setup of a formal eCRF. Where free text was entered, these elements were removed by coercion at curation. The missingness remained low suggesting this was not a problem. |
| 13. Database rule checks are in place to identify conflicts in data entries for related or dependent data collected in different CRFs or sources. | Not applicable |
| 14. There are mechanisms in place to enforce/ensure that time-sensitive data are entered within allotted time windows. | Not applicable- limited time-sensitive data elements. |
| 15. There is clear documentation of interdependence of CRF fields, including data entry skip logic. | Not applicable- no interdependence in this dataset |
| 16. Data collection includes fields for documenting that participants meet inclusion/exclusion criteria. | Not applicable- survey of all patients |
| 17. The data entry tool does not perform rounding or truncation of entries that might result in precision loss. | Spreadsheets did not perform rounding |
| 18. Extract/transform/load software for batch upload of data from other sources such as assay results should flag impossible and implausible values. | Not applicable: Data extracted by hand. |
| 19. Internationalisation is undertaken in a robust manner, and translation and cultural adaption of concepts (e.g. assessment tools) follow best practice. | This was not considered but checked at curation. It was possible to convert all units to a standard unambiguously except for International Normalised Ratio (INR): This data item was therefore dropped from the analysis (prothrombin time provides a proxy) |
| 20. Data collection methods are documented | This was not possible, but data elements were self-explanatory. |

| | |
|---|---|
| in study manuals that are sufficiently detailed to ensure the same procedures are followed each time. | |
| 21. All personnel responsible for entering data receive training and testing on how to complete the CRF. | All personnel were clinically trained and should have a good grasp of these standard data from ICU. |
| 22. The CRF/eCRF is easy to use and include a detailed description of the data collection guidelines and how to complete each field in the form. They are pilot-tested in a rigorous pre-specified and documented process until reliability and validity are demonstrated. | Not performed / deemed necessary. |
| 23. Data collectors are tested and provided with feedback regarding the accuracy of their performance across all relevant study domains. | Not performed / not possible. |
| 24. Data collection that requires specific content expertise is carried out by trained and/or certified investigators. | All personnel were clinically trained and should have a good grasp of these standard data from ICU. |
| 25. Assessors are blinded to treatment allocation or predictor variables where appropriate and such blinding is explicitly recorded. | Not applicable: All patients recruited. |
| 26. There is a clear audit chain for any data processing that takes place after entry, and this should have a mechanism for version control if it changes. | All curation and analysis have been done programmatically and an audit trail exists. |
| 27. Data are provided in a form that is unambiguous to researchers. | Self-explanatory data |
| 28. For physiological data, the methods of measurement and units are defined for all sites. | See point 19 |
| 29. Imaging acquisition techniques are standardised (e.g. magnetic resonance imaging). | Not applicable (no imaging involved) |
| 30. Biospecimen preparation techniques are standardised. | Results obtained from standard hospital assays which are QC in accordance with standard clinical laboratory practice |
| 31. Biospecimen assay accuracy, precision, repeatability, detection limits, quantitation limits, linearity and range are defined. Normal ranges are determined for each assay. | Results obtained from standard hospital assays which are QC in accordance with standard clinical laboratory practice. |
| 32. There is automated entry of the results of biospecimen samples. | Manual data entry was used for this multi-centre site as automation was not possible. |
| 34. Proxy responses for factual questions (such as employment status) are allowed in order to maximise completeness. | Not applicable (data only study) |
| 35. Automated variable transformations are documented and tested before implementation and if modified. | Documented programmatically |
| 36. There is centralised monitoring of the completeness and consistency of information during data collection. | Not feasible but curation was centralised after collection. |
| 37. Individual data elements should be checked for missingness. This should be done against pre-specified skip-logic/missingness masks. This should be performed throughout the study data acquisition period to give accurate “real time” feedback on completion status. | Done and presented in the supplementary material. Missingness was low (generally <<10%) except for a small number of variables. The small number of variables with high missingness |
| 38. Systematic and timely measures are in place to assure ongoing data accuracy. | Single time-point study so no ongoing monitoring. |
| 39. Source data validation procedures are in place to check for agreement between the original data and the information recorded in the database. | Not possible due to time / resource constraints. |
| 40. Reliability checks have been performed on variables that are critical to research hypotheses, to ensure that information from multiple sources is consistent. | Analysis was stratified by site and variability was acceptable. |
| 41. Scoring of tests is checked. Scoring is | Not applicable. |

| | |
|---|---|
| performed automatically where possible. | |
| 42. Data irregularities are reported back to data collectors in a systematic and timely process. There is a standard operating procedure for data irregularities to be reported back to the data collectors and for documentation of the resolution of the issue. | Not performed due to time / resource constraints but |
| 43. Known/emergent issues with the data dictionary are documented and reported in an accessible manner. | Data will be held by study team. |
| 44. The version lock-down of the database for data entry is clearly specified. | Data will be held by study team who maintain an audit trail. |
| 45. A plan for ongoing curation and version control is specified. | Not applicable. |
| 46. A comprehensive data dictionary is available for end users. | Data elements are self-explanatory. Data element list / dictionary provided in this supplement. |

## S3. Data quality: completeness

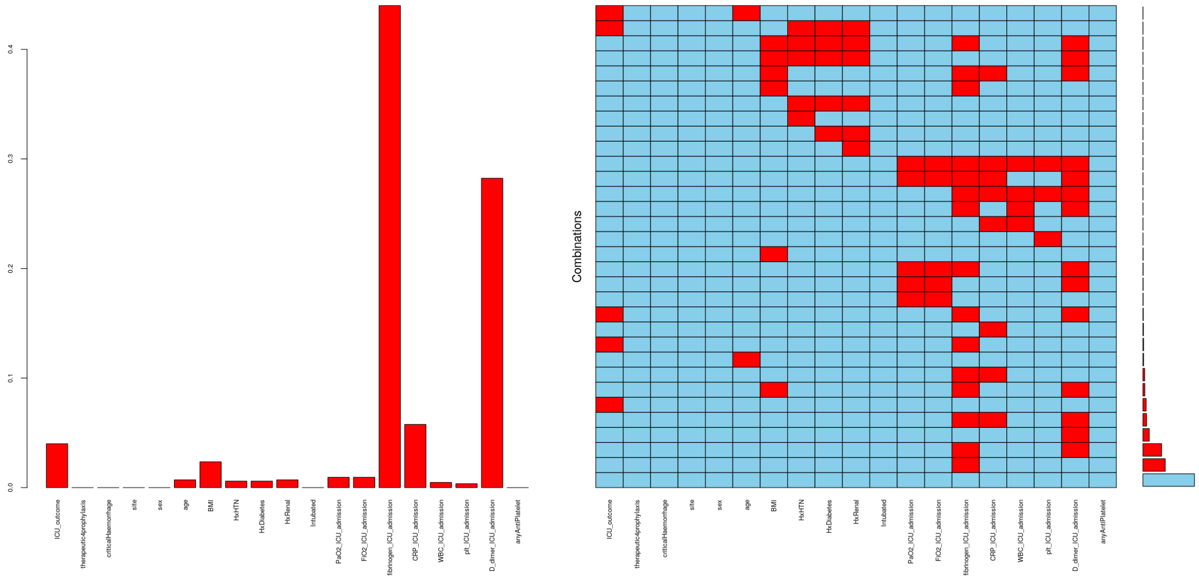


**Figure S1.** Distribution of missing data before multiple imputation. Missingness was generally very low (<<10%). The largest missingness was for fibrinogen at ICU admission (missing ~50% of cases).

## S4. Distributions for enhanced prophylaxis group

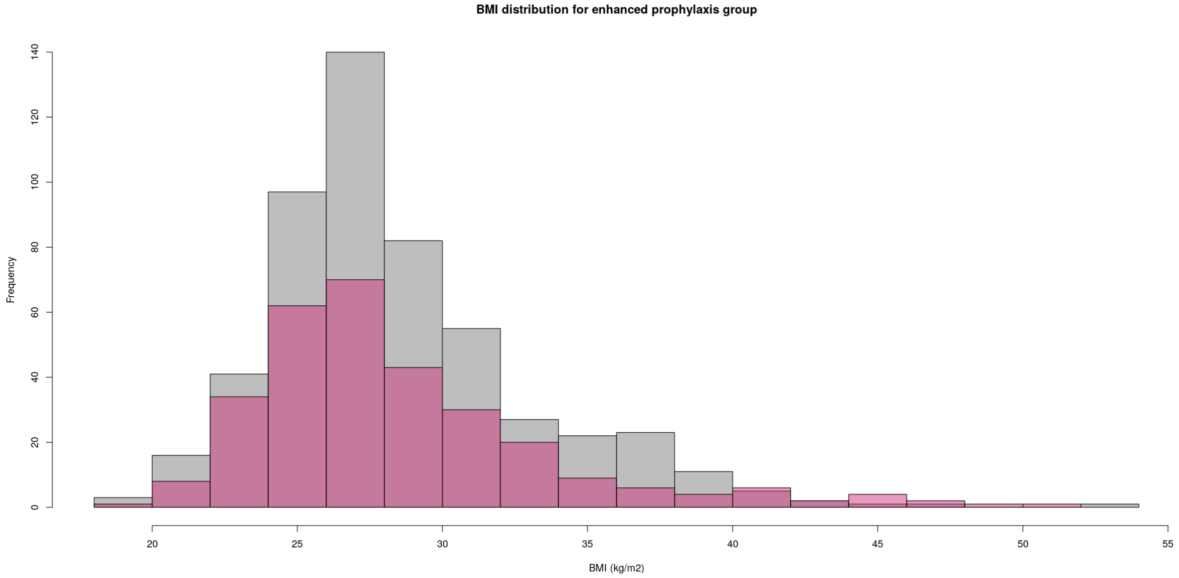


**Figure S2.** Distribution of body mass index (BMI) in the enhanced prophylaxis group (purple) compared to the rest of the patients (grey) showing no difference in median BMI (Wilcoxon rank sum *p*=1).

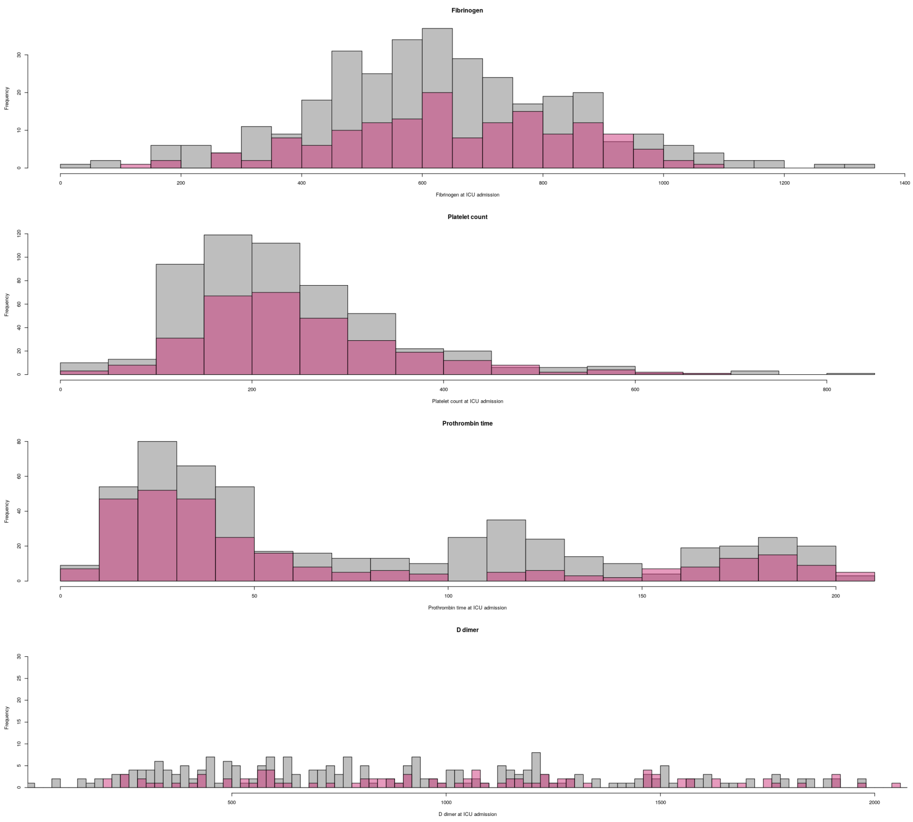


**Figure S3.** Distribution of ICU admission fibrinogen, platelet count, prothrombin time and D-dimer in the enhanced prophylaxis group (purple) compared to the rest of the patients (grey) showing no difference in median values (Wilcoxon rank sum all *p*=1).

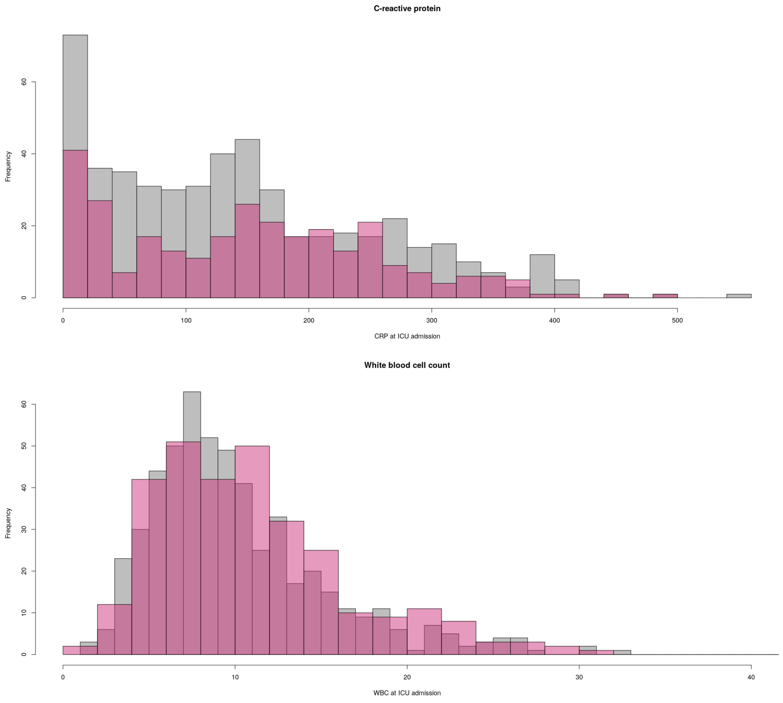


**Figure S4.** Distribution of ICU admission C-reactive protein and white blood cell count in the enhanced prophylaxis group (purple) compared to the rest of the patients (grey) showing no difference in median values (Wilcoxon rank sum all *p*=1).

## S5. Propensity score sensitivity analysis

A sensitivity analysis was conducted for the propensity score models excluding patients receiving therapeutic heparin for non-prophylaxis indications to ensure results were not biased in favour of the enhanced prophylaxis group by including patients with significant thromboembolic complications in the control group. The results were found to be qualitatively identical with the same associations as in the main models.

| Term | Effect size (log odds) | *p*-value | 95% CI | |
|---|---|---|---|---|
| (Intercept) | 0.391 | 0.4 | -0.444 | 1.23 |
| Use of 'enhanced' (therapeutic) prophylaxis | 0.663 | 0.01 | 0.157 | 1.17 |
| **Age (years)** | **-12.5** | **<0.0001** | **-16.3** | **-8.68** |
| **BMI** | **-1.9** | **0.005** | **-3.22** | **-0.571** |
| History of hypertension | -0.0618 | 0.8 | -0.62 | 0.496 |
| History of diabetes | 0.00873 | 0.9 | -0.695 | 0.712 |
| History of renal disease | -1.14 | 0.07 | -2.36 | 0.0776 |
| Intubated | 2.18 | 0.0005 | 0.955 | 3.4 |
| D-dimer at ICU_admission | -1990 | 0.61 | -9600 | 5630 |
| P/F ratio at ICU admission | 21.8 | 0.1 | -4.35 | 48 |
| CRP at ICU admission | -34.6 | 0.09 | -74.9 | 5.71 |
| Fibrinogen at ICU_admission | 25.8 | 0.53 | -54.7 | 106 |
| **Platelet count at_ICU_admission** | **42.5** | **0.01** | **8.31** | **76.7** |
| WBC at ICU admission | -1.27 | 0.2 | -3.11 | 0.578 |
| Antiplatelet agent use | 0.373 | 0.3 | -0.318 | 1.06 |

**Table S1.** Mixed effects, generalised linear model for ICU survival matched for propensity for use of 'enhanced' prophylaxis but **excluding all patients anticoagulated for non-prophylaxis indications.** Effect sizes are unscaled log-odds (positive indicates survival benefit). Abbreviations: BMI, body mass index; ICU, intensive care unit; P/F partial pressure of oxygen/inspired fraction of oxygen; CRP, C-reactive protein; WBC, white blood cells. Significant associations are identical to model presented in the main text.

| Term | Effect size (log odds) | *p*-value | 95% CI | |
|---|---|---|---|---|
| (Intercept) | -3.08 | <0.0001 | -4.18 | -1.98 |
| Use of ‘enhanced’ (therapeutic) prophylaxis | 0.189 | 0.7 | -0.68 | 1.06 |
| Age (years) | -3.62 | 0.1 | -8.21 | 0.975 |
| BMI | -0.52 | 0.6 | -2.73 | 1.69 |
| History of hypertension | -0.351 | 0.5 | -1.26 | 0.555 |
| History of diabetes | -0.452 | 0.5 | -1.82 | 0.921 |
| History of renal disease | -0.157 | 0.9 | -2.28 | 1.97 |
| Intubated | -0.749 | 0.4 | -2.47 | 0.969 |
| D-dimer at ICU_admission | -3690 | 0.6 | -16900 | 9540 |
| P/F ratio at ICU admission | 8.75 | 0.6 | -27 | 44.5 |
| CRP at ICU admission | 13.8 | 0.7 | -50.6 | 78.3 |
| Fibrinogen at ICU_admission | -68.2 | 0.3 | -204 | 67.6 |
| Platelet count at_ICU_admission | -10.4 | 0.7 | -62.3 | 41.6 |
| **WBC at ICU admission** | **2.31** | **0.02** | **0.458** | **4.17** |
| Antiplatelet agent use | 0.71 | 0.2 | -0.393 | 1.81 |

Table S2. Mixed effects, generalised linear model for occurrence of ‘critical haemorrhage’ (intracranial haemorrhage or haemorrhage requiring transfusion matched for propensity for use of ‘enhanced’ prophylaxis but **excluding all patients anticoagulated for non-prophylaxis indications**.. Effect sizes are unscaled log-odds (positive indicates associate with increased risk of critical haemorrhage). Abbreviations: BMI, body mass index; ICU, intensive care unit; P/F partial pressure of oxygen/inspired fraction of oxygen; CRP, C-reactive protein; WBC, white blood cells. Significant associations are identical to model presented in the main text.

## S6 Data dictionary

**Demographics**
Town
Hospital
Pt name
Pt surname
Date of birth
Gender
Weight [kg] (est.)
Height [cm] (est.)

**Past medical hystory**
Hx of hypertension
Hx of diabetes
Hx of renal failure
Hx of cardiac failure
Hx of liver failure

**Clinical course and outcomes**
Date onset of symptoms
Date of admission icu
Date of first intubation
Date of discharge icu
Icu outcome mode of death
Renal failure
Thromboembolic events
Haemorragic events

**Blood exams on ICU admission**
Total wbc (ICU adm)
Lymph (ICU adm)
Plt (ICU adm) pt (ICU adm)
D-dimer (ICU adm)
Fibrinogen (ICU adm)
CRP (ICU adm)
Troponin (ICU adm)
Creatinin (ICU adm)
$PaO_2$ (ICU adm)
$FiO_2$ (icu adm)

**Worst blood during ICU stay**
White blood cells (highest)
White blood cells (lowest)
Lymphocytes (lowest)
Pletelets (highest)
Pletelets (lowest)

PT (highest)
PT (lowest)
D-dimer (highest)
CRP (highest)
Troponin (highest)
Creatinine (highest)
AST (highest)
$PaO_2$ (lowest)
$FiO_2$ (highest)

**Antiplatelet therapy**
Antiplatelet therapy
Antiplatelet dose [mg] (mode)
Antiplatelet days
Antiplatelet started on admission
Antiplatelet complications

**DVT prophylaxis**
DVT prophylaxis
Dose (mode) [mg or units]
Prophylaxis days
Started on ICU admission
DVT prophylaxis complications

**Therapeutic anticoagulation**
Therapeutic anticoagulation
Started on admission
Indication for therapeutic anticoagulation
Therapeutic anticoagulation complications
